# Obesity negatively impacts corneal nerves in patients with diabetes mellitus

**DOI:** 10.1186/s40662-025-00433-5

**Published:** 2025-04-23

**Authors:** Calesta Hui Yi Teo, Chang Liu, Mingyi Yu, Isabelle Xin Yu Lee, Ansa Anam, Ching-Yu Cheng, Yadana Htunwai, Jasmine Shimin Koh, Suresh Rama Chandran, Yu-Chi Liu

**Affiliations:** 1 Yong Loo Lin School of Medicine, National University of Singapore, Singapore, Singapore; 2 Corneal and Refractive Surgery Group, Singapore Eye Research Institute, The Academia, 20 College Road, Level 6, Singapore, 169856 Singapore; 3 Department of Ophthalmology, MTI Khyber Teaching Hospital, Peshawar, Pakistan; 4 Department of Cornea, Singapore National Eye Centre, Singapore, Singapore; 5 Department of Ophthalmology, Yong Loo Lin School of Medicine, National University of Singapore, Singapore, Singapore; 6 Centre for Innovation and Precision Eye Health, Yong Loo Lin School of Medicine, National University of Singapore, Singapore, Singapore; 7 Department of Neurology, National Neuroscience Institute, Tan Tock Seng Hospital Campus, Singapore, Singapore; 8 Department of Endocrinology, Singapore General Hospital, Singapore, Singapore; 9 Ophthalmology and Visual Sciences Academic Clinical Program, Duke-NUS Medical School, Singapore, Singapore; 10 Department of Ophthalmology, National Taiwan University, Taipei, Taiwan

**Keywords:** Corneal nerves, Corneal epithelium, Obesity, Body mass index, Diabetes mellitus

## Abstract

**Background:**

To investigate the relationship between obesity and corneal nerve metrics in patients with type 2 diabetes mellitus (DM).

**Methods:**

This cross-sectional study included a total of 385 healthy controls and 663 patients with DM. Metrics for corneal nerve and epithelial cells were evaluated using in-vivo confocal microscopy (IVCM). Corneal nerve and epithelial cell parameters were quantified and compared between patients with and without obesity and across six different body mass index (BMI) categories. Multivariable regression analyses were conducted to determine the association between corneal nerve metrics and BMI in patients with DM.

**Results:**

Of the DM participants, 162 (25.4%) had obesity. Compared to the non-obese group, patients with obesity had significantly lower corneal nerve fiber density (CNFD, *P* < 0.0001), corneal nerve fiber length (CNFL, *P* = 0.002), and corneal nerve branch density (CNBD, *P* = 0.005). Analyses across different BMI categories showed a progressive decline in corneal nerve parameters including CNFD (*P* < 0.0001), CNFL (*P* < 0.0001), CNBD (*P* < 0.0001), corneal nerve fiber total branch density (*P* = 0.003), corneal nerve fiber area (*P* = 0.04), and corneal nerve fiber fractal dimension (*P* = 0.02) with increasing obesity severity. Multivariable regression analyses demonstrated that lower CNFD (β: − 0.21, 95% CI: − 0.29 to − 0.13, *P* < 0.0001), shorter CNFL (β: − 0.12, 95% CI: − 0.17 to − 0.07, *P* < 0.0001), and lower CNBD (β: − 0.17, 95% CI: − 0.30 to − 0.04, *P* = 0.01) were significantly associated with BMI after adjusting for confounders. There were no significant differences in the corneal epithelial parameters between the obese and non-obese groups.

**Conclusions:**

General obesity, specifically higher BMI, adversely affects corneal nerve health in individuals with DM. Evaluation of corneal nerves and resultant keratopathy should be considered in patients with DM and concomitant obesity.

**Supplementary Information:**

The online version contains supplementary material available at 10.1186/s40662-025-00433-5.

## Background

Chronic hyperglycemia not only exerts detrimental effects on peripheral nerves, but also on corneal nerve fibers, which is increasingly recognized as a sensitive surrogate marker for diabetic peripheral neuropathy [1]. Over the years, in-vivo confocal microscopy (IVCM) has emerged as a reliable, non-invasive, and real-time method to quantitatively analyze the corneal subbasal nerve plexus. Emerging evidence highlights that corneal nerve abnormalities not only precede the clinical onset of diabetic peripheral neuropathy but also correlate with its severity [2]. Beyond that, alterations in small nerve fibers have shown to be implicated in other central and peripheral neurodegenerative diseases, including Parkinson’s disease [3] and chronic inflammatory demyelinating polyradiculoneuropathy [4]. The corneal nerve network plays a vital role in regulating corneal homeostasis and provides neurotrophic support for maintaining the integrity of the ocular surface and epithelial barrier [5]. In hyperglycemia, several interrelated pathological processes, including the accumulation of advanced glycation end products, activation of protein kinase C, synergistically lead to the loss and damage of corneal nerve fibers [6]. This in turn diminishes the release of key neurotransmitters from corneal nerves, resulting in poor regeneration of epithelial cells and compromised epithelial function [7]. Consequently, these changes culminate in neuropathic corneas and manifest clinically as a spectrum of complications, ranging from dry eye, reduced corneal sensitivity, neuropathic corneal pain, persistent epithelial defects, to more severe sequelae, including neurotrophic keratopathy or sight-threatening corneal perforations [6].

Obesity is characterized by an excessive or abnormal accumulation of fat or adipose tissue in the body. Beyond its well-established association with systemic complications, such as cardiovascular diseases, diabetes mellitus (DM), and hyperlipidemia, it has also surfaced as a substantial risk factor for peripheral neuropathies [8, 9]. The extensive body of research elucidating the connections between DM and peripheral neuropathy has contributed to our understanding of the complex neuropathic mechanisms involved. While DM is a well-known risk factor for peripheral neuropathies, recent findings have proposed obesity as a separate risk factor, independent of glycemic status, and could be a strong independent driver for peripheral neuropathies. Studies have shown that increased adiposity, particularly central obesity, correlates with peripheral neuropathy independent of glycemic control. For instance, increased waist circumference has been associated with a higher risk of neuropathy, even among normoglycemic individuals [10–12]. A cross-sectional study observed a higher prevalence of neuropathy in normoglycemic patients with obesity compared to lean controls, along with a notable reduction in intradermal nerve fiber density in the former group [13]. Additional investigations also found symptomatic distal symmetric polyneuropathy to be more common in those with central obesity, regardless of glycemic status [14, 15].

While research has increasingly shed light on diabetes-related ocular complications, there are limited studies investigating the direct impact of raised BMI or obesity on corneal nerve metrics, and the majority of the existing knowledge stems from animal models. Hargrave et al. found that mice fed with a high-fat diet exhibited early and significant losses in corneal sensitivity and nerve density compared to their normal diet counterparts, with subsequent restoration of corneal nerve structure and function following a reversal to normal diet [16]. Similarly, another mice study reported a reduction in corneal sensitivity and nerve density in diet-induced obesity models, regardless of glycemic status [17]. A clinical study of 20 patients further demonstrated that patients with obesity presented with corneal nerve loss and subclinical neuropathy [18]. Bariatric surgery, a surgical intervention for obesity, has also shown positive effects in promoting corneal nerve regeneration [18, 19]. Specifically, a recent study showed that corneal nerve microstructure, corneal sensitivity, and peripheral neuropathy symptoms improved following bariatric surgery [20], further supporting the link between obesity and corneal neuropathy.

Despite evidence that normoglycemic obesity independently increases the risk for peripheral neuropathy, the association between obesity and diabetic corneal neuropathy (DCN) has not been reported. Therefore, we aim to investigate if obesity poses a risk for DCN by exploring the relationship between BMI and corneal nerve parameters in a large population of patients with diabetes.

## Methods

### Study population

This is a cross-sectional study conducted at the Singapore Eye Research Institute, with participants recruited from September 2022 to June 2023. The study included patients with type 2 DM who were age- and sex-matched with non-diabetic healthy controls. DM was diagnosed if the patient met one or more of the following criteria: (1) fasting blood glucose ≥ 7.0 mmol/L, or (2) 2-h 75-g oral glucose tolerance test ≥ 11.0 mmol/L, or (3) symptoms of hyperglycemia with random blood glucose ≥ 11.0 mmol/L. The duration of DM was recorded. Patients with pre-existing or concomitant ocular conditions, such as active ocular surface diseases, prior ocular or corneal surgery, history of corneal pathology such as keratitis, > 6 months of contact lens wear, and > 2 years use of anti-glaucoma medications, that may affect the corneal nerve status, were excluded. Controls were recruited if they met the following criteria: (1) no history of DM, (2) no known systemic or ocular diseases, and (3) no previous ocular surgery affecting corneal nerve parameters. Participants’ demographic data, including sex, race, and age were recorded. Specifically, the participants’ height and weight were measured using a stadiometer, and BMI was calculated. The participants were divided into two groups based on their BMI (kg/m^2^): (1) non-obese: BMI < 30, and (2) obese: BMI ≥ 30. They were also stratified into six categories based on their BMI (kg/m^2^): (1) underweight: BMI < 18.5, (2) normal: 18.5 ≤ BMI < 25, (3) overweight: 25 ≤ BMI < 30, (4) class I obesity: 30 ≤ BMI < 35, (5) class II obesity: ≤ 35 BMI < 40, and (6) class III obesity: BMI > 40. Blood tests were done to assess their diabetes [hemoglobin A1c (HbA1c)] and lipid status [cholesterol, high-density lipoprotein (HDL) cholesterol, low-density lipoprotein (LDL) cholesterol, and triglycerides levels]. Approval for the study was granted by the Institutional Review Board of SingHealth (reference number 2022/2046), and the study was conducted in accordance with the Declaration of Helsinki. All patients provided informed consent for the study.

### In-vivo confocal microscopy (IVCM) image acquisition

Laser scanning IVCM Heidelberg Retina Tomography III (Rostock Cornea Module, Heidelberg Engineering GmbH, Germany) was performed for all patients. Both corneas of each patient were examined with our published protocol [21]. Briefly, scans were first obtained at the central area of the patient’s cornea. The patient was then instructed to fixate on the flashing light in various directions with their contralateral eye to stabilize the scanning view, and the superior, inferior, nasal, and temporal region (each at an approximate distance of 3 mm away from the corneal apex) were scanned.

### Corneal subbasal nerve plexus image analysis

An experienced ophthalmologist (C.L.), who was masked to the study, selected five images from each of the following areas: the central cornea and the four corneal quadrants. These 25 representative micro images with the best-focused corneal subbasal nerve plexus, featuring well-defined, densely anastomosed, thin, and linear structures with homogenous reflectivity and Y-shaped or H-shaped branches, were selected. ACCMetrics automatic software (University of Manchester, Manchester, UK) was used to quantify the seven corneal nerve parameters [22, 23]: (1) corneal nerve fiber density (CNFD: the number of fibers/mm^2^, each frame area was 0.16033 mm^2^), (2) corneal nerve fiber length (CNFL: total length of fiber mm/mm^2^), (3) corneal nerve branch density (CNBD: the number of branch points on the main fibers/mm^2^), (4) corneal nerve fiber total branch density (CTBD: total number of branch points/mm^2^), (5) corneal nerve fiber area (CNFA: total nerve fiber area mm^2^/mm^2^), (6) corneal nerve fiber width (CNFW: average nerve fiber width mm/mm^2^), and (7) corneal nerve fiber fractal dimension (CFracDim: metric of corneal nerve morphology to measure the spatial loss of nerves).

### Corneal sensitivity assessment

A handheld Cochet-Bonnet esthesiometer (Luneau Ophthalmologie, Paris, France) containing a thin retractor nylon monofilament was used to assess the patients’ corneal sensitivity. The 6.0-cm adjustable nylon filament was applied perpendicularly to the central and peripheral four quadrants of the subject’s cornea, starting from 6.0 cm and progressively reducing in 5-mm steps until the first response occurred (total scales 0–30 cm for five areas).

### Corneal epithelial cells image analysis

Fifteen best images, encompassing a large area of epithelial cell coverage and enhanced visibility of the borders of the epithelial cells from the central cornea and each of four quadrants were selected for analysis. The AIConfocal Rapid Image Evaluation System Software (ARIES; ADCIS, Saint-Contest, Basse-Normandic, France) was used to quantify three corneal epithelial parameters: (1) density (μm^−2^), (2) average cell size (μm^2^), and (3) cell circularity [4π(area/perimeter^2^)] using published protocol [24]. A perfect circle is denoted with circularity = 1, with a decreasing circularity value indicating greater deviation. The mean values of the 15 images of each eye were obtained for each parameter.

### Statistical analysis

Analyses were performed using STATA software (version 17, STATACorp, College Station, TX). All normally distributed data are expressed as mean ± standard deviation, while non-normally distributed data are expressed as median (interquartile range). The normality of data distribution was assessed using the Kolmogorov–Smirnov test. For the comparison between obese and non-obese groups, an independent *t-*test was applied for normally distributed data, and the Mann–Whitney U test was used on non-normally distributed data, with the average of the right and left eye being used. For the differences between obesity categories, one-way analysis of variance (ANOVA) and Bonferroni post-hoc tests were used on normally distributed data, and the Kruskal–Wallis H test on non-normally distributed data. Pearson correlation analysis was also performed to evaluate the relationship between corneal nerve parameters and corneal epithelial parameters. Multivariable regression analysis was used for variables that had *P* values less than 0.1 in the univariate regression analysis to determine the association between corneal parameters and BMI. A backward stepwise regression analysis was performed for factor selection after assessing the collinearity among the parameters, including confounders. Adjustments for potential confounders, including HbA1c [25], DM duration [26], cholesterol and triglycerides levels [27], were included in the multivariable model. *P* values less than 0.05 indicates statistical significance.

## Results

### Demographics and clinical characteristics

A total of 385 healthy subjects (770 eyes), and 663 patients with type 2 DM (1326 eyes) were included. Of the healthy controls, the mean age was 57.4 ± 14.8 years, and 54.0% of the participants were male. Three hundred and four (79.0%) participants were Chinese, 39 (10.1%) were Indian, 17 (4.4%) were Malay, and 25 (6.5%) were of other races. Supplemental Table 1 shows the demographic and clinical characteristics of healthy controls. Of the participants with DM, the mean age was 58.4 ± 11.9 years, and 53.5% of the participants were male. Three hundred and seventy-seven (56.9%) participants were Chinese, 203 (30.6%) were Indian, 49 (7.4%) were Malay, and 34 (5.2%) were of other races. The median of BMI in the DM population was 26.5 (23.7–30.0) kg/m^2^, and the distribution was right-skewed with a unimodal distribution (Supplemental Fig. 1), consistent with the global obesity epidemic [28]. Of them, 25.4% of them had obesity (i.e., BMI ≥ 30 kg/m^2^) with a mean BMI of 33.6 ± 3.1 kg/m^2^, and the non-obese group had a mean BMI of 24.9 ± 2.9 kg/m^2^ (*P* < 0.0001). While the 24.9 kg/m^2^ in the non-obese group is close to the threshold for being overweight (BMI ≥ 25 kg/m^2^), it is still considerably lower than the BMI definition for obesity (BMI > 30 kg/m^2^). The mean DM duration in the population was 15.7 ± 9.8 years, and there was no significant difference in the duration of DM between the obese and non-obese groups (*P* = 0.72). There were also no significant differences between the two groups when comparing all the other variables. Table 1 shows the demographic and clinical characteristics of the study participants with DM.

**Table 1:** Demographics and clinical characteristics of study participants with diabetes

Parameter	Obese	Non-obese	*P* value
BMI (kg/m^2^)	33.6 ± 3.1	24.9 ± 2.9	** < 0.0001**
Sex			
Male	85 (52.5%)	256 (53.8%)	0.77
Female	77 (47.5%)	220 (46.2%)	
Race			
Chinese	78 (48.2%)	285 (59.9%)	0.38
Indian	57 (35.2%)	138 (29.0%)	
Malay	18 (11.1%)	29 (6.1%)	
Others	9 (5.6%)	24 (5.0%)	
Age (years)	56.9 ± 11.6	58.7 ± 12.3	0.13
Duration of DM (years)	15.4 ± 8.8	15.7 ± 10.2	0.72
HA1c (%)	8.3 ± 1.6	8.1 ± 1.5	0.17
Lipid Status (mmol/L)			
Total Cholesterol	4.3 ± 1.2	4.4 ± 1.1	0.39
LDL Cholesterol	2.3 ± 1.0	2.4 ± 0.9	0.45
HDL Cholesterol	1.2 ± 0.4	1.3 ± 0.3	0.21
Triglycerides	1.8 ± 1.1	1.8 ± 1.2	0.97
Estimated glomerular Filtration Rate (mL/min/1.73 m^2^)	88.8 ± 29.1	89.9 ± 30.1	0.71

*BMI* = body mass index; *DM* = diabetes mellitus; *HbA1c* = hemoglobin A1c; *LDL* = low-density lipoprotein; *HDL* = high-density lipoprotein

Student’s *t-*test was used. *P* values in bold indicate statistical significance

**Fig. 1:**
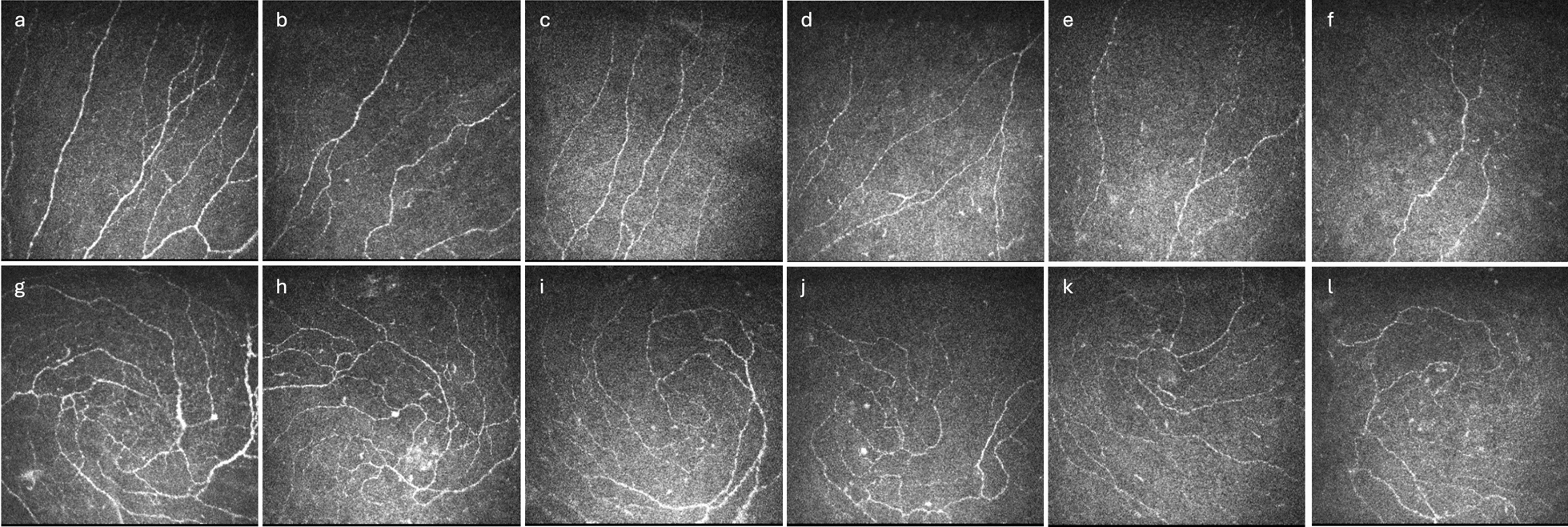
In-vivo confocal microscopy (IVCM) images of corneal nerves in each body mass index (BMI) category. Representative IVCM images of corneal nerves and inferior whorl across different BMI categories: (**a**) underweight, (**b)** normal, (**c**) overweight, (**d**) class I obesity, (**e**) class II obesity, and (**f**) class III obesity. Representative IVCM images illustrating inferior whorl morphology across different stages of BMI categories: (**g**) underweight, (**h**) normal, (**i**) overweight, (**j**) class I obesity, (**k**) class II obesity, and (**l**) class III obesity. As BMI severity increases, there is a progressive decrease in corneal nerve fiber density, length, branch density, total branch density, area, and fractal dimension

### Patients with diabetes and concomitant obesity have significantly worse corneal nerve parameters

No significant differences in corneal nerve parameters were observed between ethnic groups. Patients with diabetes were found to have significantly worse corneal nerve parameters compared to the healthy controls (all *P* < 0.005, Supplemental Table 2). In the DM group, patients with obesity had significantly lower CNFD (obese: 10.5 ± 5.0 fibers/mm^2^ vs. non-obese: 12.4 ± 5.3 fibers/mm^2^, *P* < 0.0001), CNFL (obese: 8.0 ± 2.6 mm/mm^2^ vs. non-obese: 8.6 ± 3.3 mm/mm^2^, *P* = 0.002), and CNBD (obesity: 10.0 ± 7.5 branch points on main fibers/mm^2^ vs. non-obese: 11.6 ± 8.7 branch points on main fibers/mm^2^, *P* = 0.005) as compared to the patients without obesity. Patients with obesity also had borderline significantly lower CTBD (obese: 18.1 ± 11.1 branch points/mm^2^ vs. non-obese: 19.7 ± 12.3 branch points/mm^2^, *P* = 0.06). There were no significant differences in the corneal sensitivity and epithelial cells parameters between both groups (Table 2). Significant differences in the corneal nerve and epithelial parameters between the obese and non-obese group were not observed in healthy controls (Supplemental Table 1).

**Table 2:** Comparisons of corneal nerve metrics between obese and non-obese groups with diabetes

Parameter	Obese	Non-obese	*P* value
Corneal nerve fiber parameters			
CNFD (no. of fibers/mm^2^)	10.5 ± 5.0	12.4 ± 5.3	** < 0.0001**
CNFL (total length of fibers mm/mm^2^)	8.0 ± 2.6	8.6 ± 3.3	**0.002**
CNBD (no. of branch points on main fibers/mm^2^)	10.0 ± 7.5	11.6 ± 8.7	**0.005**
CTBD (total no. of branch points/mm^2^)	18.1 ± 11.1	19.7 ± 12.3	0.06
CNFA (total nerve fiber area mm^2^/mm^2^)	0.0040 ± 0.0013	0.0040 ± 0.0016	0.93
CNFW (average nerve fiber width mm/mm^2^)	0.022 ± 0.001	0.022 ± 0.001	0.41
CFracDim	1.388 ± 0.050	1.389 ± 0.047	0.69
Corneal sensitivity (cm)	28.3 ± 2.6	28.6 ± 2.5	0.10
Corneal epithelial cell parameters			
Density (μm^–2^)	0.008 ± 0.001	0.008 ± 0.002	0.14
Size (μm^2^)	127.5 ± 10.7	125.8 ± 14.0	0.13
Circularity	0.717 ± 0.017	0.718 ± 0.021	0.68

*CNFD* = corneal nerve fiber density; *CNBD* = corneal nerve branch density; *CNFL* = corneal nerve fiber length; *CTBD* = corneal nerve fiber total branch density; *CNFA* = corneal nerve fiber area; *CNFW* = corneal nerve fiber width; *CFracDim* = corneal nerve fiber fractal dimension

Student’s *t-*test was used. *P* values in bold indicate statistical significance

### Corneal nerve status deteriorated with increasing BMI

**Fig. 2:**
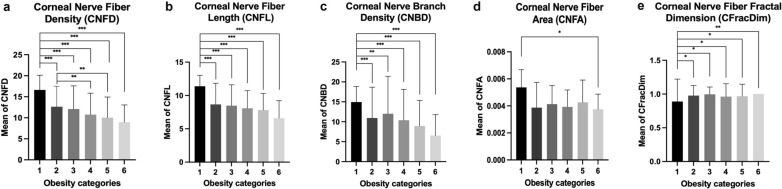
Subgroup analysis of corneal nerve parameters across different body mass index (BMI) categories. Bar charts showing the comparisons of corneal nerve parameters across various BMI categories: (**a)** corneal nerve fiber density (CNFD, number of fibers/mm^2^), (**b)** corneal nerve fiber length (CNFL, total length of fibers mm/mm^2^), (**c)** corneal nerve branch density (CNBD, number of branch points on main fibers/mm^2^), (**d)** corneal nerve fiber area (CNFA, total nerve fiber area mm^2^/mm^2^), and (**e)** corneal nerve fractal dimension (CFracDim). The y-axis represents the mean values of each corneal nerve parameter, while the x-axis are the different BMI (kg/m^2^) categories: (1) underweight: BMI < 18.5, (2) normal: 18.5 ≤ BMI < 25, (3) overweight: 25 ≤ BMI < 30, (4) class I obesity: 30 ≤ BMI < 35, (5) class II obesity: 35 ≤ BMI < 40, and (6) class III obesity: BMI > 40. *, *P* < 0.05, **, *P* < 0.005, ***, *P* < 0.0001

**Fig. 3:**
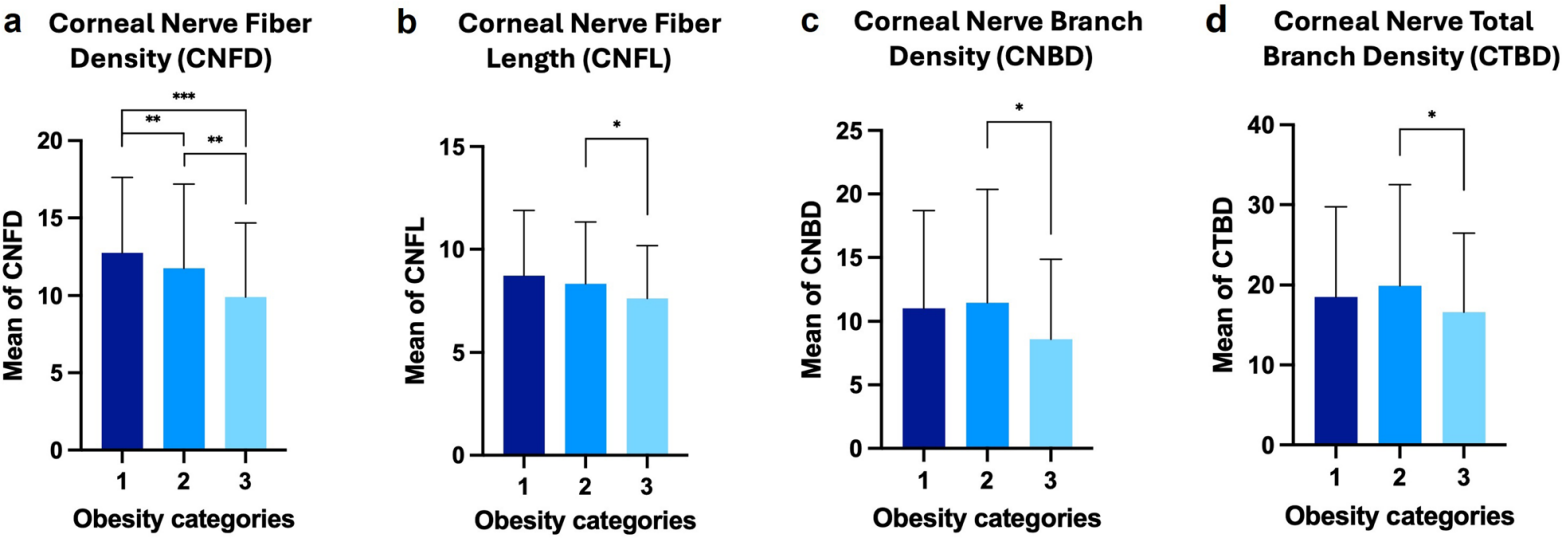
Subgroup analysis of corneal nerve parameters across different body mass index (BMI) categories combined. Bar charts showing the comparisons of corneal nerve parameters across various BMI categories: (**a)** corneal nerve fiber density (CNFD, number of fibers/mm^2^), (**b)** corneal nerve fiber length (CNFL, total length of fibers mm/mm^2^), (**c)** corneal nerve branch density (CNBD, number of branch points on main fibers/mm^2^), (**d)** corneal nerve total branch density (CTBD, number of branch points on main fibers/mm^2^). The y-axis represents the mean values of each corneal nerve parameter, while the x-axis displays different BMI (kg/m^2^) categories: (1) underweight (BMI < 18.5) + normal (18.5 ≤ BMI < 25); (2) overweight (25 ≤ BMI < 30) + class I obesity (30 ≤ BMI < 35); (3) class II obesity (35 ≤ BMI < 40) + class III obesity (BMI > 40). *, *P* < 0.05, **, *P* < 0.005, ***, *P* < 0.0001

Subgroup analyses were conducted to examine the differences in corneal nerve parameters across different obesity categories: underweight (n = 9), normal BMI (n = 221), overweight (n = 425), class I obesity (n = 121), class II obesity (n = 34), and class III obesity (n = 7). Deteriorations in corneal nerve parameters were observed with the increase in obesity severity (Fig. 1). As BMI severity increases, there is a noticeable decrease in corneal nerve metrics, including CNFD (*P* < 0.0001), CNFL (*P* < 0.0001), CNBD (*P* < 0.0001), CTBD (*P* = 0.003), CNFA (*P* = 0.04) and CFracDim (*P* = 0.02) (Table 3 and Fig. 2). When we merged the obesity categories (underweight + normal; overweight + class I obesity; class II and III obesity), deterioration of CNFD (*P* = 0.04), CNFL (*P* = 0.01), CNBD (*P* = 0.03), CTBD (*P* = 0.04) across these categories remains significant (Table 4 and Fig. 3).

**Table 3:** Difference in corneal nerve parameters between different BMI categories in patients with diabetes

Parameter	Underweight (n = 9)	Normal (n = 221)	Overweight (n = 425)	Class I obesity (n = 121)	Class II obesity (n = 24)	Class III obesity (n = 7)	ANOVA *P* value
CNFD (no. of fibers/mm^2^)	16.6 ± 3.4	12.6 ± 4.9	12.1 ± 5.5	10.8 ± 5.1	10.1 ± 4.9	10.8 ± 4.8	** < 0.0001**
CNFL (total length of fibers mm/mm^2^)	11.4 ± 1.6	8.7 ± 3.2	8.5 ± 3.1	8.1 ± 2.7	7.9 ± 2.5	7.7 ± 2.8	** < 0.0001**
CNBD (no. of branch points on main fibers/mm^2^)	14.9 ± 3.8	11.0 ± 7.7	12.0 ± 0.4	10.4 ± 7.8	8.9 ± 6.4	11.4 ± 12.3	** < 0.0001**
CTBD (total no. of branch points/mm^2^)	28.6 ± 14.2	18.3 ± 11.1	20.6 ± 13.1	18.6 ± 11.5	17.1 ± 9.9	20.4 ± 17.1	**0.003**
CNFA (total nerve fiber area mm^2^/mm^2^)	0.0053 ± 0.0013	0.0039 ± 0.0018	0.0040 ± 0.0014	0.0041 ± 0.0013	0.0039 ± 0.0013	0.0033 ± 0.0015	**0.04**
CNFW (average nerve fiber width mm/mm^2^)	0.0226 ± 0.0090	0.0219 ± 0.0011	0.0221 ± 0.0013	0.0221 ± 0.0011	0.0221 ± 0.0012	0.0223 ± 0.0013	0.11
CFracDim	1.43 ± 0.02	1.39 ± 0.04	1.40 ± 0.05	1.39 ± 0.05	1.39 ± 0.04	1.36 ± 0.06	**0.02**
Corneal sensitivity (cm)	28.5 ± 1.3	28.5 ± 2.3	28.8 ± 2.8	28.4 ± 2.9	28.2 ± 1.9	27.5 ± 2.0	0.24

*CNFD* = corneal nerve fiber density; *CNBD* = corneal nerve branch density; *CNFL* = corneal nerve fiber length; *CTBD* = corneal nerve fiber total branch density; *CNFA* = corneal nerve fiber area; *CNFW* = corneal nerve fiber width; *CFracDim* = corneal nerve fiber fractal dimension

One-way analysis of variance (ANOVA) was used. *P* values in bold indicate statistical significance

**Table 4:** Difference in corneal nerve parameters between combined BMI categories in patients with diabetes

Parameter	Underweight + Normal (n = 230)	Overweight + Class I obesity (n = 546)	Class II + III obesity (n = 31)	ANOVA *P* value
CNFD (no. of fibers/mm^2^)	12.7 ± 4.9	11.6 ± 5.4	9.9 ± 4.7	**0.04**
CNFL (total length of fibers mm/mm^2^)	8.4 ± 3.1	8.3 ± 3.0	7.6 ± 2.5	**0.01**
CNBD (no. of branch points on main fibers/mm^2^)	11.1 ± 7.6	11.4 ± 8.9	8.5 ± 6.2	**0.03**
CTBD (total no. of branch points/mm^2^)	18.4 ± 11.1	19.9 ± 12.6	16.6 ± 9.8	**0.04**
CNFA (total nerve fiber area mm^2^/mm^2^)	0.0039 ± 0.0017	0.0040 ± 0.0013	0.0038 ± 0.0013	0.30
CNFW (average nerve fiber width mm/mm^2^)	0.0219 ± 0.0110	0.0221 ± 0.0012	0.0221 ± 0.0012	0.08
CFracDim	1.39 ± 0.03	1.39 ± 0.04	1.38 ± 0.04	0.07
Corneal sensitivity (cm)	28.5 ± 2.2	28.7 ± 2.8	28.1 ± 1.9	0.15

*CNFD* = corneal nerve fiber density; *CNBD* = corneal nerve branch density; *CNFL* = corneal nerve fiber length; *CTBD* = corneal nerve fiber total branch density; *CNFA* = corneal nerve fiber area; *CNFW* = corneal nerve fiber width; *CFracDim* = corneal nerve fiber fractal dimension

One-way analysis of variance (ANOVA) was used. *P* values in bold indicate statistical significance

Specifically, CNFD and CNFL were significantly lower in the normal, overweight, class I obesity, class II obesity, and class III obesity groups compared to those who were underweight (all *P* < 0.0001). CNFD was also significantly lower in patients with class I obesity and class II obesity, compared with those in the normal group (*P* = 0.001 and *P* = 0.005, respectively). CNBD was significantly lower in the overweight, class I, II, and III obesity groups, compared to the underweight group (all *P* < 0.0001), as well as in the normal compared with the underweight group (*P* = 0.001). Moreover, class III obesity individuals had a significantly lower CNFA than those who were underweight (*P* = 0.05). Lastly, CFracDim was significantly lower in the normal (*P* = 0.02), overweight (*P* = 0.02), class I obesity (*P* = 0.03), class II obesity (*P* = 0.04), and class III obesity (*P* = 0.004) groups compared with the underweight group. Specifically, in the merged categories, CNFD was significantly lower in the class II + class III obesity groups when compared with the underweight + normal group (*P* < 0.0001), in the overweight + class I obesity group compared to the underweight + normal group (*P* = 0.004), as well as in the class II + class III obesity group compared to overweight + class I obesity group (*P* = 0.025). In addition, patients with class II + class III obesity had significantly lower CNFL than patients who were overweight + class I obesity (*P* = 0.02). Moreover, those classified as class II + class III obesity had significantly lower CNBD (*P* = 0.02) and CTBD (*P* = 0.04) compared to those who were classified as overweight + class I obesity. These findings underscore a progressive deterioration in corneal nerve quantity and quality as BMI increases. Detailed subgroup post-hoc analyses of the original and combined BMI categories are shown in Figs. 2 and 3, respectively.

### Associations between corneal nerve parameters and BMI

**Fig. 4:**
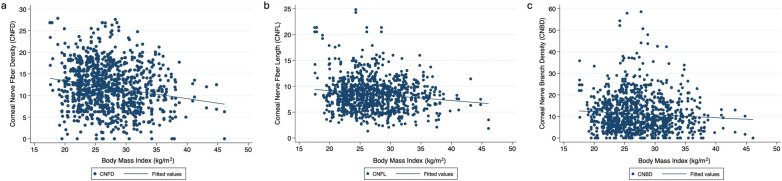
Scatter plots of body mass index (BMI) and significant corneal nerve parameters. Scatter plots demonstrating significant associations between BMI and corneal nerve parameters among the study participants: (**a)** corneal nerve fiber density (CNFD, number of fibers/mm^2^), (**b)** corneal nerve fiber length (CNFL, total length of fibers mm/mm^2^), and (**c)** corneal nerve branch density (CNBD, number of branch points on main fibers/mm^2^). The plots show a decline in corneal nerve parameters with increasing BMI. The x-axis represents the BMI values, and the y-axis represents the values of the respective corneal nerve parameters

In the univariate regression model, all the nerve parameters exhibited a deteriorating trend with increasing BMI (Fig. 4). Lower CNFD (β: − 0.21, 95% CI: − 0.28 to − 0.14, *P* < 0.0001), shorter CNFL (β: − 0.10, 95% CI: − 0.14 to − 0.06, *P* < 0.0001), and lower CNBD (β: − 0.14, 95% CI: − 0.25 to − 0.03, *P* = 0.01) were significantly associated with BMI (Table 5). Multivariate regression analyses demonstrated that a lower CNFD (β: − 0.21, 95% CI: − 0.29 to − 0.13, *P* < 0.0001), shorter CNFL (β: − 0.12, 95% CI: − 0.17 to − 0.07, *P* < 0.0001), and lower CNBD (β: − 0.17, 95% CI: − 0.30 to − 0.04, *P* = 0.01) remained significantly associated with BMI after adjusting for confounders (Table 5).

**Table 5:** Associations between body mass index and corneal nerve parameters in patients with diabetes

	Univariate analysis			Multivariate analysis*		
Parameter	β	95% CI	*P* value	β	95% CI	*P* value
CNFD (no. of fibers/mm^2^)	− 0.22	− 0.28 to − 0.14	** < 0.0001**	− 0.21	− 0.29 to − 0.13	** < 0.0001**
CNFL (total length of fibers mm/mm^2^)	− 0.10	− 0.14 to − 0.06	** < 0.0001**	− 0.12	− 0.17 to − 0.07	** < 0.0001**
CNBD (no. of branch points on main fibers/mm^2^)	− 0.14	− 0.25 to − 0.03	**0.01**	− 0.17	− 0.30 to − 0.04	**0.01**
CTBD (total no. of branch points/mm^2^)	− 0.09	− 0.24 to 0.07	0.27	− 0.14	− 0.31 to 0.04	0.12
CNFA (total nerve fiber area mm^2^/mm^2^)	− 0.002	− 0.0001 to 0.0001	0.87	− 0.010	− 0.0001 to 0.0001	0.89
CNFW (average nerve fiber width mm/mm^2^)	0.02	0.02 to 0.02	0.09	0.02	0.02 to 0.02	0.12
CFracDim	− 0.0005	− 0.0010 to 0.0002	0.15	− 0.0006	− 0.0010 to 0.0001	0.27

Univariate and multivariate analyses were used

^*^Adjusted for HbA1c, duration of diabetes, cholesterol, and triglyceride levels

*P* values in bold indicate statistical significance

*CNFD* = corneal nerve fiber density; *CNBD* = corneal nerve branch density; *CNFL* = corneal nerve fiber length; *CTBD* = corneal nerve fiber total branch density; *CNFA* = corneal nerve fiber area; *CNFW* = corneal nerve fiber width; *CFracDim* = corneal nerve fiber fractal dimension

### Associations between BMI and corneal epithelial cells

There were no significant differences in the corneal epithelial cell density, size and circularity between the obese and non-obese groups (Table 2). In both the univariate and multivariate regression models, corneal epithelial cell parameters were not found to be significantly associated with BMI. When analyzing the correlations between corneal epithelial parameters and corneal nerve parameters, a statistically significant positive correlation was observed between CNFW and epithelial circularity (r = 0.011, *P* = 0.003), coupled with a negative correlation with epithelial cell density (r = − 0.076, *P* = 0.05). CFracDim demonstrated a positive correlation with epithelial cell size (r = 0.082, *P* = 0.04).

## Discussion

In this study, those with obesity were found to have significantly lower CNFD, CNFL, and CNBD when comparing with their non-obese counterparts. As BMI increased, all corneal nerve parameters showed a marked deterioration. After adjustment for confounding factors in the multivariate analyses, lower CNFD, CNFL, and CNBD remained significantly associated with BMI. These data suggest that obesity, particularly a raised BMI, plays a pivotal role in the development of DCN. These insights have potential therapeutic ramifications, highlighting the importance of mitigating and controlling obesity-related risk factors for corneal nerve health.

In line with our findings, Gulkas et al. reported moderate and significant negative correlations between BMI and CNFD (r = − 0.55, *P* < 0.001), CNFL (r = − 0.50, *P* = 0.003), CNBD (r = − 0.64, *P* < 0.001) and CfracDim (r = − 0.39, *P* = 0.006) [29]. They also presented a significant positive correlation between BMI and CNFW (r = 0.42, *P* = 0.003) [29]. Moreover, individuals with obesity, regardless of glycemic status, had significantly reduced CNFD, CNFL, CTBD, and CNFA compared with those who are non-obese [29]. Here, further stratification into the different BMI categories revealed a progressive deterioration in corneal nerve parameters as BMI increases. Specifically, patients with class I and II obesity had significantly lower CNFD compared to those with normal BMI. When we collapsed the six obesity groups, deterioration of CNFD, CNFL, CNBD, CTBD across these combined categories remains significant. The results from our multivariate analyses indicate that for every five units increase in BMI, i.e., exacerbation in one BMI severity category, CNFD decreased by 1.05 nerve fibers/mm^2^, CNFL decreased by 0.60 mm/mm^2^, and CNBD decreased by 0.85 branch points/mm^2^, respectively, based on the β values. Given that the average area of the cornea is approximately 122 mm^2^, we can further quantify the extent of nerve fiber loss associated with the increase in BMI. For instance, a shift from normal weight (i.e., BMI of 24 kg/m^2^) to the overweight category (i.e., BMI of 29 kg/m^2^) would result in a loss of approximately 1.05 fibers/mm^2^ × 122 mm^2^ = 128.1 corneal nerve fibers.

Up until the last few decades, the consensus has been that chronic hyperglycemia is the main driver for neuropathy. It is now better understood that other components of metabolic syndrome, specifically obesity, may potentially play a significant role in the development of diabetic neuropathy [10, 13, 14, 30]. While obesity alone has been linked to the development of polyneuropathy, in the absence of diabetes [10], its impact on corneal neuropathy remains unexplored. Data have suggested that small unmyelinated axons, such as the corneal nerve plexus, may be disproportionately more predisposed to injury from obesity and hypertriglyceridemia, compared to larger myelinated fibers, which are more susceptible to hyperglycemic damage [31]. The pathophysiology of obesity-associated small fiber neuropathy is complex and thought to arise from mitochondrial dysfunction, oxidative stress, extracellular protein glycation, and neuroinflammation, in addition to glucotoxicity effects [30]. The link between obesity and DCN could also be attributed to metabolically driven cardiovascular risk factors, such as hypertension, hyperlipidemia, and inflammation, leading to degenerative processes within small nerve fibers.

In obesity, alteration of lipid and glycemic profiles are almost always concomitant. Hence, independent pathways from merely obesity are difficult to rule out, and are likely to cross-talk with lipid signaling and glucotoxic events. In our non-DM healthy controls, we did not observe a significant difference in corneal nerve parameters in the obese group, compared to the non-obese group. Longer diabetes duration and poorer glycemic control may have contributed to poorer corneal nerve structures through multiple interconnected mechanisms [6]. Briefly, the accumulation of advanced glycation end-products leads to segmental demyelination and impaired axonal transport, impairing nerve regeneration [6]. Excess glucose metabolism via the polyol pathway also leads to osmotic stress, oxidative damage, and reduced Na^+^/K^+^ ATPase activity, which disrupts nerve conduction [6]. Additionally, mitochondrial dysfunction and depletion of neurotrophic factors, such as ciliary neurotrophic factor and nerve growth factor further impair corneal nerve regeneration and sensory function, contributing to corneal nerve fiber loss and dysfunction in DM [6]. These key metabolic disruptions driving neuropathy in DM are not present in non-DM individuals, likely explaining the lack of significant impairment in non-DM subjects. While obesity may still induce inflammation, these effects alone may not cause the same degree of corneal nerve damage observed in DM. Numerous studies have shown that obesity together with dyslipidemia and hypertriglyceridemia are highly correlated with neuropathy [8, 31, 32]. The available data also points to the involvement of metabolic syndrome in the development of neuropathy in patients with obesity [30]. A recent study using lipidomics and metabolomics found that specific subclasses of metabolites distinguished individuals with obesity by peripheral neuropathy status, independent of glycemia [33]. Some investigations have also proposed mechanistic pathways through which obesity may exacerbate nerve damage, including chronic inflammation, oxidative stress, and metabolic dysregulation [33, 34]. Concomitant dyslipidemia, particularly elevated triglycerides, may contribute to corneal nerve damage by promoting further inflammation and neuronal oxidative stress, accelerating corneal nerve degeneration. Experimental findings suggest that long chain fatty acids, and inflammatory mediators can penetrate the blood-nerve barrier and activate neurogenic inflammation. These neurons subsequently produce vasoactive signals that increase vascular permeability and recruit adaptive immune cells [35, 36]. Obesity has been shown to activate resident macrophages, dendritic cells, and inflammatory mediators [37], with changes in these cell population being linked to reduced nerve density and delayed nerve regeneration [38, 39]. Furthermore, obesity-induced oxidative stress may result in altered corneal biomechanics, contributing to the development of corneal neuropathy [40]. These findings may also underscore the distinct metabolic and pathogenic pathways involved in corneal nerve damage in individuals with DM and concomitant obesity or obesity alone.

Notably, a recent animal study found that reduced corneal sensitivity was correlated with weight and adiposity, independent of age or diet, and this was associated with a significant increase in the rate of neuronal-epithelial cell fusion [41]. Our study did not observe a significant difference in corneal sensitivity between the obese and non-obese groups, nor between the BMI categories. These can be attributed to the fact that clinical function and nerve morphology do not always align. Corneal sensitivity can be present even in the absence of visible nerve pathology, and conversely, nerve pathology may exist without corresponding alterations in clinical functionality [42]. A study on DM demonstrated that the loss of corneal nerve fiber bundles precedes sensory impairment. Mild-to-moderate neuropathy showed decreased corneal nerve innervation, yet only severe neuropathy was associated with significantly reduced sensitivity [43], suggesting that structural changes in the corneal nerves may occur before clinical functional changes. This inconsistency highlights that the current tools used for assessing corneal sensitivity may be too imprecise and lack the necessary sensitivity to identify early or subtle alterations in nerve function. A larger degree of neuronal damage may then be needed before any clinically appreciable deterioration in corneal sensitivity could be observed.

It is speculated that neuronal-epithelial cell fusion may contribute to nerve reorganization and the loss of corneal innervation [41]. Here, we found a positive correlation between CNFW and epithelial circularity, along with a negative correlation with epithelial cell density. These might indicate that altered nerve function or impaired trophic support (i.e., more swollen corneal nerves) leads to a disruption of the corneal epithelial structure. Furthermore, the positive correlation between CFracDim and epithelial cell size might suggest an adaptive change to maintain trophic support for nerve fibers in the face of neuronal degeneration or spatial loss of nerve fibers. However, while these were statistically significant, the statistical correlations were weak. We did not see significant differences in the corneal epithelial parameters between the obese and non-obese groups. One possible explanation is the continuous regenerative properties of corneal epithelium and its structural resilience [44]. The processes of cell proliferation, migration, and desquamation help maintain epithelial integrity [45], potentially mitigating the negative impact induced by obesity. Additionally, neuropathy-related changes in the epithelium may only become apparent in more severe cases [46, 47]. Further studies, including longitudinal studies that track temporal changes in both corneal nerve and epithelial parameters will provide insights into the long-term effects of obesity.

While rigorous glucose control is widely acknowledged to be the cornerstone treatment for diabetic neuropathy, including corneal neuropathy, this emerging evidence suggests that the development of DCN may also involve non-glucose signaling, and that dietary and weight management may have a role in reversing corneal neuropathy. A recent study demonstrated that participants treated with glucose-lowering medications linked to weight gain experienced more severe neuropathy and greater corneal nerve loss over time, compared to those treated with medications associated with weight loss [48], further supporting our findings. Additionally, gastric bypass has also been proven to drastically improve peripheral neuropathy symptoms score [49] and corneal nerve metrics [18] in patients with concomitant obesity and DM.

This study has several limitations. First, this is a cross-sectional study. As such, longitudinal weight changes and its impact on corneal nerve and epithelial changes were not assessed and can be included in future studies. Secondly, BMI thresholds for obesity vary based on ethnicity. As our study included both Asian and Caucasian populations, we employed the standard thresholds for obesity classification recommended for Caucasian populations. This approach might not fully account for ethnic-specific variations in obesity definitions. Lastly, past medical history, medication data, smoking history, physical activity level, and socioeconomic status were not included due to reliance on self-reported history, which is subject to recall bias. Additionally, research exploring the underlying mechanisms linking obesity to DCN through tear molecular studies could be valuable. Longitudinal and interventional clinical studies could also examine whether weight loss programs, alongside glycemic control, offer protective effects against DCN progression. These investigations may pave the way for targeted interventions aimed at mitigating the detrimental effects of obesity on corneal nerve function.

## Conclusion

This study provides compelling evidence that higher BMI adversely affects corneal nerve health in individuals with DM. It suggests that elevated BMI may independently contribute to corneal nerve fiber damage. Therefore, regular evaluation and monitoring of corneal nerves, as well as resultant neurotrophic keratopathy, could be considered for patients with DM and concomitant obesity. Our findings underscore the need for comprehensive management strategies that prioritize not only glycemic control but also optimizing weight management to effectively mitigate the risk and progression of corneal nerve damage.

## Supplementary Information

Additional file 1: Figure 1. Histogram of body mass index (BMI) distribution among the study population. The histogram is right-skewed and unimodal, indicating a higher prevalence of elevated BMI within the study population. The x-axis represents the BMI values, and the y-axis shows the frequency of individuals. Table 1. Demographic characteristics and IVCM parameters between obese and non-obese groups in healthy controls. Table 2. In-vivo confocal microscopy parameters between healthy controls and participants with type 2 diabetes mellitus.

## Data Availability

The datasets used and/or analyzed during the current study are available from the corresponding author upon reasonable request.

## Abbreviations

DM: Diabetes mellitus
DCN: Diabetic corneal neuropathy
BMI: Body mass index
HbA1c: Hemoglobin A1c
HDL: High-density lipoprotein
LDL: Low-density lipoprotein
IVCM: In-vivo confocal microscopy
CNFD: Corneal nerve fiber density
CNBD: Corneal nerve branch density
CNFL: Corneal nerve fiber length
CTBD: Corneal nerve fiber total branch density
CNFA: Corneal nerve fiber area
CNFW: Corneal nerve fiber width
CFracDim: Corneal nerve fiber fractal dimension
