# Supplementary material for: Obesity negatively impacts corneal nerves in patients with diabetes mellitus
Source: Eye Vis (Lond). 2025 Apr 23;12:17. doi: 10.1186/s40662-025-00433-5 (PMC12016176; doi:10.1186/s40662-025-00433-5)
Supplement: Supplementary file 1 — Additional file 1: Figure 1. Histogram of body mass index (BMI) distribution among the study population. The histogram is right-skewed and unimodal, indicating a higher prevalence of elevated BMI within the study population. The x-axis represents the BMI values, and the y-axis shows the frequency of individuals. Table 1. Demographic characteristics and IVCM parameters between obese and non-obese groups in healthy controls. Table 2. In-vivo confocal microscopy parameters between healthy controls and participants with type 2 diabetes mellitus. [file 40662_2025_433_MOESM1_ESM.docx]

**Supplemental Figure 1. Histogram of body mass index (BMI) distribution among the study population.** The histogram is right-skewed and unimodal, indicating a higher prevalence of elevated BMI within the study population. The x-axis represents the BMI values, and the y-axis shows the frequency of individuals.

**
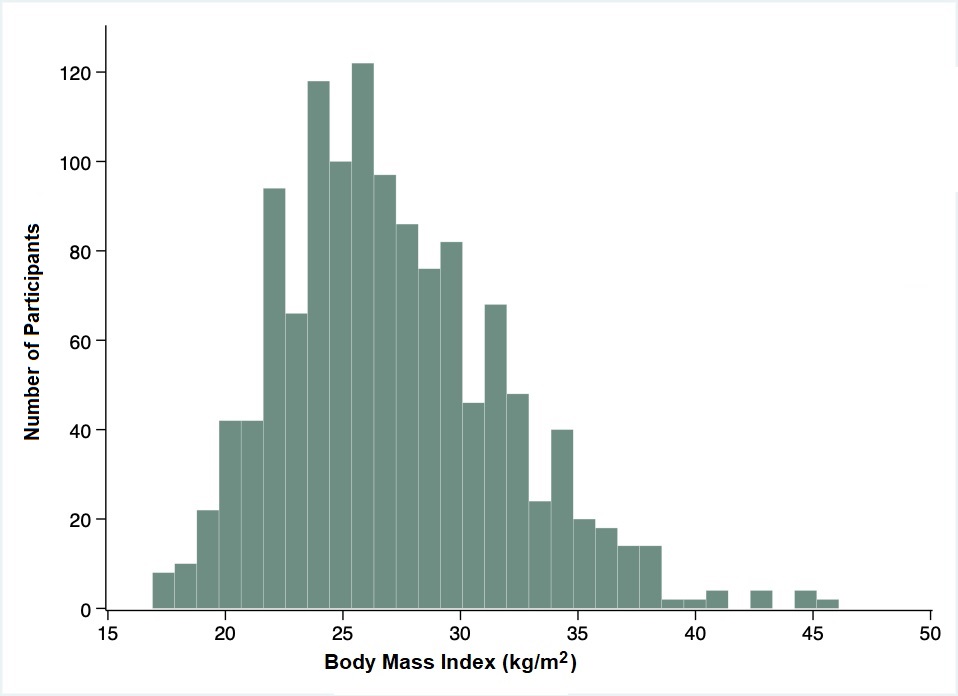
**

**Supplemental Table 1.** Demographic characteristics and IVCM parameters between obese and non-obese groups in healthy controls.

| **Parameter** | **Obesity** | **Non-Obese** | ***P* value** |
| --- | --- | --- | --- |
| Demographics | | | |
| BMI (kg/m^2^) | 33.5 ± 4.9 | 23.6 ± 3.1 | **< 0.0001** |
| Sex |  |  |  |
| Males | 18 (51.4%) | 139 (51.7%) | 0.98 |
| Females | 17 (48.6%) | 130 (48.3%) |  |
| Race |  |  |  |
| Chinese | 25 (71.4%) | 231 (85.9%) | 0.07 |
| Indian | 7 (20.0%) | 23 (8.6%) |  |
| Malay | 1 (2.9%) | 10 (3.7%) |  |
| Others | 2 (5.7%) | 5 (1.9%) |  |
| Age (years) | 57.4 ± 13.7 | 63.1 ± 10.3 | **0.004** |
| Corneal nerve fiber parameters | | | |
| CNFD (no. of fibers/mm^2^) | 13.1 ± 5.2 | 13.5 ± 5.4 | 0.71 |
| CNFL (total length of fibers mm/mm^2^) | 8.8 ± 2.7 | 8.8 ± 2.8 | 0.99 |
| CNBD (no. of branch points on main fibers/mm^2^) | 12.3 ± 9.4 | 12.3 ± 9.6 | 0.99 |
| CTBD (total no. of branch points/mm^2^) | 21.3 ± 12.6 | 21.3 ± 13.9 | 0.99 |
| CNFA (total nerve fiber area mm^2^/mm^2^) | 0.0043 ± 0.0014 | 0.0042 ± 0.0015 | 0.64 |
| CNFW (average nerve fiber width mm/mm^2^) | 0.0220 ± 0.0009 | 0.0210 ± 0.001 | 0.71 |
| CFracDim | 1.40 ± 0.04 | 1.40 ± 0.05 | 0.53 |
| Corneal epithelial cell parameters | | | |
| Density (μm^–2^) | 0.0080 ± 0.0006 | 0.0080 ± 0.003 | 0.65 |
| Size (μm^2^) | 127.3 ± 8.9 | 126.4 ± 9.2 | 0.63 |
| Circularity | 0.71 ± 0.014 | 0.72 ± 0.015 | 0.08 |

BMI = body mass index; CNFD = corneal nerve fiber density; CNFL = corneal nerve fiber length; CNBD = corneal nerve branch density; CTBD = corneal nerve fiber total branch density; CNFA = corneal nerve fiber area; CNFW = corneal nerve fiber width; CFracDim = corneal nerve fiber fractal dimension.

P values in bold indicate statistical significance.

**Supplemental Table 2.** In-vivo confocal microscopy (IVCM) parameters between healthy controls and participants with type 2 diabetes mellitus.

| **Parameter** | **Healthy controls** | **Type 2 diabetes mellitus** | ***P* value** |
| --- | --- | --- | --- |
| Corneal nerve fiber parameters | | | |
| CNFD (no. of fibers/mm^2^) | 13.7 ± 5.5 | 11.9 ± 5.2 | **< 0.0001** |
| CNFL (total length of fibers mm/mm^2^) | 9.1 ± 2.9 | 8.4 ± 3.0 | **< 0.0001** |
| CNBD (no. of branch points on main fibers/mm^2^) | 12.7 ± 9.6 | 11.2 ± 8.5 | **0.0005** |
| CTBD (total no. of branch points/mm^2^) | 22.2 ± 14.0 | 19.3 ± 12.2 | **< 0.0001** |
| CNFA (total nerve fiber area mm^2^/mm^2^) | 0.0043 ± 0.0020 | 0.0040 ± 0.0020 | **0.0001** |
| CNFW (average nerve fiber width mm/mm^2^) | 0.0215 ± 0.0010 | 0.0220 ± 0.0010 | **< 0.0001** |
| CFracDim | 1.40 ± 0.05 | 1.39 ± 0.05 | **< 0.0001** |
| Corneal sensitivity | 28.2 ± 3.4 | 28.6 ± 2.6 | 0.01 |
| Corneal epithelial cell parameters | | | |
| Density (μm^−2^) | 0.0081 ± 0.0020 | 0.0082 ± 0.0020 | 0.27 |
| Size (μm^2^) | 127.6 ± 10.3 | 126.3 ± 13.2 | 0.09 |
| Circularity | 0.716 ± 0.020 | 0.717 ± 0.020 | 0.30 |

CNFD = corneal nerve fiber density; CNFL = corneal nerve fiber length; CNBD = corneal nerve branch density; CTBD = corneal nerve fiber total branch density; CNFA = corneal nerve fiber area; CNFW = corneal nerve fiber width; CFracDim = corneal nerve fiber fractal dimension.

*P* values in bold indicate statistical significance.
